# Death from the Inhalation of Chloroform, in Cincinnati

**Published:** 1848-04

**Authors:** 


*Death from the Inhalation of Chloroform.*—Report of the principal facts connected with a fatal case of chloroform inhalation, which occurred in Cincinnati, on the 23d of February, 1848:

*General history.*—The subject of the following report, Mrs. Martha G. Simmons, was at the time of her death thirty-five years and ten months old. Her husband states that she generally enjoyed excellent health; sometimes she was 'nervous,' and suffered occasionally with neuralgic pains about the face and pain in the ear, apparently arising from decayed teeth. She also suffered at times from 'sick headache.' She was the mother of six children, five of whom are still living; her last accouchement occurred eight weeks previous to her death. Nothing unusual occurred, either at the time of parturition or subsequently; her health remained good, and the ordinary quantity of milk was secreted.

On the 23d of February she dined at a quarter past twelve o'clock, and after dinner walked to a dentist's, a distance of about three-fourths of a mile, for the purpose of having some roots of teeth extracted. She arrived at the dentist's fifteen minutes before three o'clock, appeared slightly flushed from the exercise of walking, but exhibited no alarm on account of inhaling the chloroform.

At three o'clock, fifteen minutes after her arrival, Mrs. S. commenced inhaling the chloroform. Mrs. Pearson and Mrs. Cross, two female friends, were present, and report the following as the events which occurred: The respiratory movements appeared to be free—chest heaving. While inhaling, *the face became pale.* At the expiration of about one minute, the instruments were applied and four roots of teeth extracted. The patient groaned and manifested what they regarded as evidences of pain while the teeth were being extracted, although she did not speak nor exhibit any other sign of consciousness. As the last root came out, which was about two minutes from the beginning of the inhalation, patient's head turned to one side, arms became slightly rigid, body drawn somewhat backwards, with a tendency to slide from the operating chair. At this instant Mrs. Pearson states that she placed her finger upon the patient's pulse, observed that it was feeble and immediately ceased to beat; respiration also ceased *about* the same time. The face, which was previously pale, now became livid, as also did the finger nails; the lower jaw dropped, and the tongue projected a little at one corner of the mouth, and the arms were perfectly relaxed. The females regarded her as being then quite dead. Efforts were made to resuscitate the patient—ammonia was applied to the nostrils, cold water dashed in the face, mustard, brandy, etc. applied. The patient was now removed from the operating chair and laid on the sofa, but she did not breathe nor exhibit any sign of life after being placed in the recumbent position.

*Statement of the dentists.*—Messrs. Meredith and Sexton, the dentists who operated in the above case, make the following statement: The patient took the chloroform vapor from Morton's inhaler; it contained a sponge (perhaps one-third filling the glass globe of 4½ inches in diameter) saturated with the liquid; to this twenty-five drops more were added when the patient began inhaling. Breathing at first slow; inhaled twelve or fifteen times, occupying from a minute to seventy-five seconds. One of the dentists thinks she remained about *ten* minutes in the operating chair, and that life was not extinct until the end of that time; the other estimates the time at *five* minutes. One says he does not know whether she breathed after being laid on the sofa or not; the other thinks she did not.

The only material difference between the statements of the females and the dentists relates to the length of time which elapsed from the beginning of the inhalation to the instant of death. The females estimate it at about two minutes, the dentists at from five to ten minutes. It is clear, however, that the patient could not have been laid on the sofa short of five or ten minutes; for one of the dentists went out to a neighboring establishment twice to procure resuscitating agents before the patient was removed from the chair, which probably occupied the time specified. But whether the patient continued to breathe during those five or ten minutes, or whether the pulse and respiration ceased at the end of two minutes, when the last tooth was extracted, as supposed by Mrs. Pearson, seems impossible positively to decide. The most that can be said is, that she died within a very short time —not exceeding *ten* and possibly at the end of *two* minutes.

*Medical aid.*—After the patient was laid on the sofa, medical aid was sought, and Dr. A. H. Baker was the first physician who arrived; this was probably 30 minutes after respiration had ceased. He immediately pronounced her dead, but proceeded to employ vigorous measures for resuscitation. The principal means employed consisted in artificial respiration, electro-magnetism, and external stimulants. Prof. Locke applied electro-magnetism, which caused active muscular contraction, but no evident effect on the heart. About an hour after the accident, Profs. Mussey and Lawson arrived, and aided in the further employment of the means above specified. Not the slightest sign of life was manifested after the arrival of Dr. Baker; the heart did not respond to the electricity, and the only change produced was some slight removal of the lividity of the countenance by the artificial respiration.

The post mortem examination was made twenty-six hours after death. Present—Drs. Mussey, Lawson, Baker and Mulford.

Examination by Dr. Lawson. Record by Dr. Mussey.

*External appearances.*—Lips livid, but face pale; bloody froth issuing from the mouth. Anterior surface of the body and limbs free from discoloration, but posteriorly the skin presented a deep livid hue. Cornea dull and flaccid, and a dull-red horizontal belt extended across each eye, corresponding to the part which was unprotected by the lids; this belt was one-tenth of an inch in diameter, and made its appearance a few hours after death. Limbs quite rigid. Abdomen distended with gas. Patient rather muscular; weight probably from 140 to 150 pounds; hair dark; eyes dark brown; temperament sanguineo-bilious.

*Brain.*—Integuments contained but little blood. On removing the upper part of the skull, a larger quantity of blood than usual flowed from the vessels of the dura mater. Superficial vessels of the brain moderately distended; two or three ounces of fluid blood, intermixed with bubbles of air, flowed from the sinuses of the dura mater. General aspect, color, and consistency of the brain normal.

*Lungs.*—Considerably but not intensely congested; crepitated freely at all points; no extravasation. Lining membrane of bronchia slightly congested, apparantly the result of recent catarrh; deeply stained by the blood. Pleura at all points highly injected; six drachms bloody serum in the right, and two ounces in the left chest.

*Heart and large Blood-Vessels.*—Pericardium contained six drachms of bloody serum. Heart flaccid, *and all its cavities entirely empty;* inner surface of both ventricles and auricles deeply stained. Aorta and pulmonary artery empty; no blood in the cava within the chest, and a very small quantity in the part which lies within the abdomen; indeed, so small was the amount that it could not be appreciated until the vessel was opened. Lining membrane of all the blood vessels deeply stained.

*Abdomen.*—One ounce and a half of bloody serum in the right hypochondrium. Stomach and intestines distended with gas. Partially digested aliment, amounting to about three gills, was found in the stomach. Liver paler than natural, arising from the absence of blood; kidneys considerably engorged. No marks of previous disease in any of the abdominal organs. Uterus and bladder normal; the former exhibited the usual condition of the organ two months after delivery.

*Blood.*—Fluid as water in every part of the body; not a coagulum was seen in any vessel. Examined with a microscope, the globules appeared altered somewhat in form; some were irregular in shape, and they seemed generally distended and more globular than is normal; they were also somewhat frag mentary, a part apparantly having been ruptured; their number seemed somewhat diminished. The color, in every part of the system, was that of dark venous blood.

*Sympathetic nerve.*—The sympathetic nerve, together with its larger ganglia, including the semilunar ganglion, presented a natural color.

*The Chloroform used.*—The specific gravity of the chloroform employed was found to be 1.3. It contained some alcohol, but upon the whole is regarded as a fair article; it was the same which the dentists had previously used in numerous cases without any unpleasant results.—*Western Lancet.*

